# A theoretical interpretation of the interaction between formal and illegal institutions in migrant labour recruitment in Italian agriculture

**DOI:** 10.3389/fsoc.2026.1937799

**Published:** 2026-10-02

**Authors:** Gaetano Martino

**Affiliations:** Department of Agricultural, Food and Environmental Sciences, University of Perugia, Perugia, Italy

**Keywords:** agricultural labour exploitation, illegal institutions, institutional interaction, Italian agri-food system, migrant workers

## Abstract

Labour exploitation is widespread in Italian agriculture. Although a substantial body of research has sought to explain these trends, further scientific investigation is still needed to understand how formal and illegal institutions interact. Institutional analysis could provide an original contribution to the study of this issue. This study adopts this perspective and seeks to address the following research question: what kinds of interactions take place among these institutions that may be associated with the emergence of the observed working and exploitative conditions? The manuscript examines migrant labour in Italian agriculture and investigates how migration regulations, migrants’ legal status, labour market dependency, and illegal recruitment practices interact in producing vulnerability and exploitation. In particular, this study contributes to the debate by shedding light on the interaction between formal and illegal institutions and by integrating them into a unified theoretical framework. Following Helmke and Levitsky (2004), this article classifies these illegal institutions and their interactions as competing in nature. The existing literature provides considerable evidence that supports this theoretical framework. However, the study has two main limitations. First, the analysis relies on a non-systematic narrative literature review. A more structured analysis could enrich the interpretation of the phenomenon. Second, the study lacks empirical research specifically designed to test the proposed theory. Future research should therefore focus on this task and further enrich the theoretical interpretation by examining, above all, the impact of institutional interactions on enterprises and agri-food value chains.

## 1 Introduction

An evident spread of labour exploitation can be observed in the agricultural sector in many countries. The risk of forced labour is high across significant sectors of the global agri-food system (Blackstone et al., 2023). Labour exploitation – the focus of this study–is a phenomenon which falls within the field of the indecent work. Scholars have highlighted the causal relationship between regulatory shortcomings and the spread of indecent work (Lewis et al., 2015; Brotherhood et al., 2024; Lenard and Straehle, 2010; Marsden, 2012; Silverman and Hari, 2016; Falco and Bartoli, 2026). Indecent labour is understood here as work performed under conditions that fall short of the International Labour Organization’s standards of decent work, including inadequate remuneration, insufficient legal protection, unsafe working environments, and restricted access to social rights, and limited opportunities for workers’ representation. Building on the work of Carchedi (2020, 2024), Guidi and Berti (2023), and Torre (2018), this study defines *agricultural labour exploitation* as a set of practices through which employers or labour intermediaries derive economic benefits from violations of labour regulations and workers’ rights, including underpayment, undeclared or irregular employment, excessive working hours, unsafe and unhealthy working conditions, restrictions on workers’ freedom, and coercive mechanisms.

Migratory processes and overtly illegal practices appear to constitute key elements of the overall picture (Perrotta and Raeymaekers, 2023; Palumbo et al., 2022; Omizzolo, 2020; Abbatecola et al., 2022; Rye and Scott, 2018; Molinari et al., 2023). This field of observation is increasingly characterised by the coexistence of workers who differ considerably in terms of protection of rights, and nationality (Carchedi, 2024). Focusing on the Italian case, as this article does, these developments can be interpreted as a further stage in the transformation of the agricultural sector, alongside the declining importance of family labour and the increase in average farm size (Istat, 2026). Italy appears to be characterised by dynamics similar to those observed in many other countries where migratory flows play a significant role. This would appear to reinforce the explanatory power of scientific interpretations informed by generalising approaches (Salvia, 2024; Corrado et al., 2024).

Nevertheless, it is worth examining certain aspects of the phenomenon more closely in order to shed light on factual circumstances that warrant further and more in-depth investigation. More specifically, this article seeks to contribute to the understanding of these complex phenomena by adopting the perspective of institutional analysis. Indeed, the widespread diffusion of illegal practices in the recruitment and management of segments of the agricultural workforce raises the analytical question of the relationship between the regulatory framework developed in Italy in recent years and the simultaneous persistence of systems of practices based on illegal rules, some of which are openly criminal. This study aims at identifying potential links between regulatory shortcomings and illegal practices and argues that this relationship should be interpreted in terms of *interactions among institutions of different kinds*. It is necessary to point out that scholars have already paid considerable attention to the relationship between public interventions, including legislation, and the exploitation of migrant workers. The focus of this article, however, is not on whether and how public interventions cause exploitation, but rather on understanding the modalities through which formal and informal institutions coexist and contribute to exploitation. This coexistence must be identified and taken into consideration because, on the one hand, it reveals the shortcomings and distortions of the current system of formal institutions and, on the other hand, it draws attention to the active role of formal institutions in shaping the characteristics of the economic system under examination. As extensively documented by empirical research (Carchedi, 2020, 2024; Carchedi and Bilongo, 2022), the operation of illegal institutions progressively spreads exploitative practices. The interaction between formal and informal institutions, therefore, has increasingly come to constitute a distinctive feature of the Italian agro-industrial system.

Institutions are systems of established and widely shared social rules that structure social interactions (Hodgson, 2025; North, 1990). The literature has conceptualised interactions among institutions in different ways. Ménard (2017) conceptualize interactions among institutions in terms of relationships among institutions operating at different levels. North (1990) developed a substantive theory explaining how formal and informal institutions shape economic change. Hodgson (2025) emphasised how the reciprocal influence of formal and informal institutions, including illegal ones, contributes to transformations in the institutional setting of a social system. Following Helmke and Levitsky (2004), this study understands institutional interaction as the coexistence of formal and informal institutions, including illegal ones, leading to different types of social outcomes. This study argues that these outcomes can be used to characterize the nature of the interaction.

As discussed in detail below, this study contends that the interaction between formal and informal institutions in the context of migrant labour recruitment in Italian agriculture is competitive in nature. This implies that the two types of institutions coexist and that informal and illegal institutions create opportunities for unlawful recruitment practices and the exploitation of migrant workers.

This study concentrates on three key elements: the regulatory framework governing both migration flows and the fight against illegal recruitment practices; the vulnerability of individuals recruited into forms of indecent work, understood in terms of limited access to rights in general and, more specifically, insufficient legal protection, which is associated with weaker bargaining power; and illegal labour intermediation. These elements are investigated and ultimately integrated into a unified framework of institutional interaction.

It should be emphasised that this article does not address the broader social or economic mechanisms that may trigger the exploitation of migrant workers in agriculture. Rather, the focus is on how the competitive interaction between formal and illegal institutions in the Italian context generates a significant acceleration in the spread of labour exploitation across numerous geographical areas and sectors of activity (Carchedi, 2020, 2024; Carchedi and Bilongo, 2022).

The article is organized as follows. Section 2 presents the research problem and the methodology adopted in the study. Section 3 illustrates and discusses the three dimensions outlined above. The conceptual interpretation in terms of institutional interaction is presented in Section 4. Section 5 concludes.

## 2 Research question and method of the study

The data on labour recruitment in agriculture account for a complex picture whose determinants picture whose determinants have to identified. To contribute to this end, this Section first illustrates the main traits of the picture and to delineate the methodology of this study.

### 2.1 Defining the research problem

The emergence of significant forms of labour exploitation linked to the recruitment, often illegal, of migrant workers in the agricultural sector has been widely recognised (Palumbo et al., 2022; Rye and Scott, 2018; Perrotta and Raeymaekers, 2023; Omizzolo, 2020). Approximately 4.7% of the global labour force is composed of migrants. In Europe, the demand for such workers is particularly high in agriculture, where seasonal labour represents a resource that cannot easily be replaced (Carchedi, 2024). Moreover, it has been estimated that around 200,000 migrants are currently employed in Italian agriculture under irregular employment conditions (Carchedi, 2024).

The 2020 Italian Agricultural Census revealed a 30% decline in the number of farms and a halving of family labour compared with 2010. Conversely, wage workers increased by 38%, reaching more than 1.27 million workers and accounted for 41.7% of the agricultural labour force, while the remaining 58.3% comprised farm holders (45.3%) and family workers (13.0%) The proportion of foreign workers has also grown considerably, exceeding 33% of the total wage workers, compared with less than 25% a decade earlier (Macrì, 2024). The national picture shows a predominance of non-EU workers, who account for nearly 60% of the foreign agricultural workforce, while EU workers represent approximately 41%. However, this distribution varies considerably across regions. In some cases, the share of non-EU workers is well above the national average, particularly in Marche, Lazio and Liguria, where non-EU labour accounts for more than 70% of the foreign agricultural workforce (Macrì, 2024).

In summary, Italian agriculture has undergone a profound transformation characterised by a reduction in the number of farms, accompanied by an increase in average farm size (Istat, 2026) and a growing reliance on wage workers. Within this workforce, foreign workers have become a predominant component, reflecting the increasing dependence of the agricultural sector on migrant labour.

Carchedi (2020, 2024); Carchedi and Bilongo (2022) has extensively documented the diffusion of migrant labour in Italian agriculture and has analyzed in detail the processes of exploitation affecting these workers. Judicial investigations conducted in Italy have shed further light on this issue. Gonnelli and Santoro (2024) show that, in the majority of investigations involving foreign victims, the exploited workers are individuals whose vulnerability stems from their irregular legal status (e.g., unauthorized entry or pending claims) in the country. Overall, such workers were involved as victims in 366 investigations concerning labour exploitation, representing 74% of the total. However, the figure relating to foreign victims holding a valid residence permit is far from negligible. More specifically, focusing on the agricultural sector, 428 cases were identified, of which 378 (88.3%) involved foreign victims. Only for 191 of these cases the study was able to ascertain the individuals’ legal status: 67 cases (35.1% of those for which the status could be established) involved legally resident individuals; 64 cases (33.5%) involved individuals without legal residence status; and 60 cases (31.4%) involved both legally resident and non-resident individuals.

An analysis of changes over time of the data reveals that, from the entry into force of Law No. 199/2016 until 2020, legally resident foreign nationals were involved in more investigations than workers without a residence permit. This trend reversed from 2021 onwards, and the predominance of cases involving irregularly resident victims became particularly marked in 2024. In summary, within the broader transformation of Italian agriculture, characterised by the growing importance of wage labour, both the increasing reliance on foreign workers and the rising presence of irregular migrants have become increasingly evident.

As illustrated below, a complex set of rules and institutions has been established; however, it appears to be accompanied by numerous illegal practices on the part of various actors. This raises the question of how these different systems of rules and institutions shape the transformation of the Italian agri-food sector. To address this issue, a specific theoretical framework and a set of related research questions are presented below.

### 2.2 Theoretical background and research question

The literature shows that the employment of migrants in Italian agriculture is characterised by the coexistence of both legal and illegal practices. This calls for an examination of the role of institutions in shaping the behaviours that give rise to such outcomes. Scholars typically distinguish between formal and informal institutions (North, 1990; Shirley, 2026; Hodgson, 2025) and recognise that illegal institutions should be considered part of the broader set of informal institutions (Hodgson, 2025). It is therefore particularly important to investigate how these different forms of institutions contribute to the configuration of migrant employment relations in Italian agriculture. In summary, although previous studies have documented the growing dependence of Italian agriculture on migrant labour and the widespread occurrence of labour exploitation, less attention has been devoted to explaining how interactions between formal and illegal institutions contribute to these outcomes. A notable gap in the literature on the exploitation of migrant workers in agriculture is the limited use of concepts derived from institutional theories and institutional analysis. The conceptual frameworks developed in these fields enable a more accurate exploration of exploitation processes, whereas many aspects remain obscure and inaccessible to inquiries that do not draw upon such analytical tools. It emerges clearly that rules of various kinds contribute to shaping the transformation of agro-industrial systems and the diffusion of exploitation practices. This, in turn, highlights the need to investigate these systems of rules directly. The benefits of pursuing these lines of investigation become all the more evident when the aim is to disentangle, without preconceived assumptions, the complex pattern revealed by empirical observation.

The present study addresses this gap by developing a theoretical framework that explains how interactions among institutions shape the emergence and persistence of labour exploitation in Italian agriculture.

The approach adopted is grounded in institutional theory and distinguishes institutions according to their nature (North, 1990; Acemoglu and Robinson, 2012; Hodgson, 2025). This study understands institutions as systems of established and prevalent social rules that structure social interaction (Hodgson, 2025). More specifically, formal institutions are constituted or maintained through legally prescribed procedures, and legal rules represent the dominant elements of their regulatory framework. Informal institutions include language, customs, rituals, and social conventions such as table manners. Most of their rules are not established through formal legislative processes, although some rules embedded within informal institutions may be illegal (Hodgson, 2025, p. 14).

Helmke and Levitsky (2004) define informal institutions as “socially shared rules, usually unwritten, that are created, communicated, and enforced outside officially sanctioned channels.” They argue that rigorous institutional analysis must take these rules into account, as informal incentives often affect formal ones in shaping political behaviour and institutional outcomes. The authors develop a fourfold typology of informal institutions based on two dimensions: the effectiveness of formal rules and the convergence of outcomes:

- *Complementary*: effective formal rules in which informal norms fill procedural gaps or enhance efficiency^1^.
- *Accommodating*: effective formal rules in which informal norms alter substantive effects without directly violating formal rules, often reconciling conflicting interests (for example, elite power-sharing arrangements).
- *Competing*: situations in which formal rules and procedures are not systematically enforced, enabling actors to ignore or violate them and thereby generating divergent outcomes. These informal institutions structure incentives in ways that are incompatible with formal rules: compliance with one set of rules requires the violation of another. Clientelism, patrimonialism, and corruption are typical examples.
- *Substitutive*: ineffective formal rules in which informal norms achieve outcomes that the formal system has failed to deliver, such as community-based justice mechanisms or forms of collective self-protection.

The literature recognises two distinct concepts of *institutional interaction*. Hodgson (2025) emphasises interactions that generate institutional change. For example, informal institutions may influence the evolution of formal institutions over time. Helmke and Levitsky (2004), by contrast, focus on the interaction between formal and informal institutions in terms of the social outcomes they produce. Their emphasis is on patterns of social interaction and behavioural consequences, whereas Hodgson’s perspective focuses primarily on the institutional framework itself and its evolution.

Given the different institutions at stake, both formal and illegal (that is, informal), the following research question emerges:

*What kinds of interaction occur between these institutions, and how can such interactions be associated with the working and exploitative conditions observed in Italian agriculture?*

This study seeks to contribute to answering this question.

To this end, a theoretical investigation is conducted with the aim of integrating, within a unified analytical framework, the roles of both formal and informal institutions in the context under examination.

### 2.3 Methodology

This study first reviews the Italian regulatory framework governing migration, agricultural employment, and illegal labour intermediation. It then compares findings from these sources in order to identify recurring institutional mechanisms linking migration regulation, migrant vulnerability, illegal recruitment, and law enforcement. On this basis, the study formulates theoretical propositions and integrates them into a unified institutional framework explaining labour exploitation in agriculture.

The method adopted combines literature analysis with the development of theoretical propositions. A theory is a set of statements concerning the relationship(s) between two or more concepts or constructs (Jaccard and Jacoby, 2019).

This study adopts a propositional style of theorising, seeking to channel theoretical ideas and arguments into a set of formally stated propositions (Cornelissen, 2017, p. 3). Before developing these propositions, it is necessary to define the concepts employed in the study. Hodgson (2019) argues that taxonomic definitions, rather than describing the full nature of an entity, identify the minimum set of properties required to distinguish one group of entities from all others.

Moreover, this study defines *illegal labour recruitment* as the unlawful hiring, recruitment, transportation, or placement of workers by intermediaries or employers who violate labour, immigration, or human rights legislation, often with the purpose of exploiting workers. This practice is frequently associated with labour exploitation, forced labour, undeclared work, and human trafficking. In Italian agriculture, illegal labour recruitment is largely based on the *caporalato* system (Perrotta and Raeymaekers, 2023; Omizzolo, 2020; Palumbo et al., 2022; Torre, 2018).

*Illegal labour intermediation*, by contrast, is defined as the unauthorised recruitment and allocation of workers by private intermediaries who profit from matching labour supply and demand while circumventing labour regulations and facilitating workers’ exploitation through coercion, vulnerability, dependency, or substandard working conditions (Carchedi, 2020, 2024; Torre, 2018; Perrotta and Raeymaekers, 2023; Omizzolo, 2020). The exploitation of foreign workers, however, is a complex phenomenon involving individuals with varying degrees of legal protection. Notably, Falco and Bartoli (2026) argue that legal status influences the subjective perception of exploitative conditions, suggesting that variations in legal status are associated with differences in working conditions.

The methodology consists of two main stages. The first involves an examination of the regulatory framework and of the literature on labour intermediation and labour exploitation, which are considered the central illegal institutions in the Italian case. The second involves the development of a theoretical framework designed to integrate the evidence emerging from the first stage of the analysis. Through these stages, three propositions are formulated and subsequently integrated into a unified framework grounded in the theory of formal and informal (illegal) institutions (North, 1990; Hodgson, 2025; Helmke and Levitsky, 2004).

Propositions are broader statements about relationships between constructs than hypotheses and typically seek to explore new theoretical territory (Cornelissen, 2017). In developing the propositions, this study follows the criteria proposed by Cornelissen (2017) and Rouse et al. (2025):

- to preserve the nature of propositions as broad statements of relationships between constructs that address novel theoretical ground, avoiding the mere reformulation of hypotheses (Cornelissen, 2017, p. 4).
- to formulate propositions parsimoniously, introducing only those statements that are strictly necessary.
- to ensure an appropriate and coherent phrasing of the propositions.
- to maintain a consistent level of analysis within each proposition.
- to identify causal agents clearly and explicitly.

The literature review was conducted using a narrative review approach (Snyder, 2019). The objective was to synthesise multiple perspectives through a critical and interpretive assessment of the literature (Sukhera, 2022, p. 414), starting from the research question that guides this study. More specifically, a meta-narrative review approach was adopted in order to understand how different narratives surrounding the phenomenon have emerged and been interpreted across distinct streams of research.

The review began with the seminal contributions of Rye and Scott (2018), Perrotta and Raeymaekers (2023), Corrado et al. (2024), and Marsden (2012). The principal databases consulted were Scopus and Web of Science. Google Scholar was also used to identify relevant literature not published in indexed journals or books, particularly with regard to the Italian case.

Two cycles of search, analysis, and interpretation were conducted. The first cycle focused on migrant labour and labour exploitation in agriculture (key search terms: *agriculture*, *migrants*, *labour exploitation*). The second cycle focused specifically on the Italian legal framework (key search terms: *“Bossi-Fini Law”* and “Law No. 199/2016”).

Based on *a priori* knowledge of the phenomenon, largely derived from the seminal studies identified at the outset of the review, a substantial number of studies were excluded. The exclusion criteria were as follows:

- Studies that had not achieved significant scholarly recognition, as indicated by a limited citation impact within the international literature.
- Studies that focused exclusively on describing legal frameworks, mechanisms, and rules^2^.

## 3 The legal framework

The first step of this study is to analyse the formal institutional framework governing labour recruitment in the Italian agricultural sector. Regulation of migration flows constitutes one of the main pillars of this framework, as it directly influences access to the labour market and the conditions under which migrant workers are employed. This section outlines the key features of Italian immigration law and examines its implications for migrant vulnerability.

### 3.1 Regulation of migration flows in Italy

#### 3.1.1 The legislative framework

The Italian immigration system is primarily governed by Legislative Decree No. 286/1998, the *Consolidated Immigration Act* (*Testo Unico sull’Immigrazione*), which remains the cornerstone of immigration law despite subsequent amendments, including the Bossi-Fini reform of 2002. The legislation regulates both migration management and migrant integration and is based on three main principles: the regulation of migration through planned admissions and the prevention of irregular migration; the protection of the rights of legally resident migrants; and the balancing of national interests with constitutional guarantees.

A defining feature of the Italian model is the regulation of migration through labour market planning. Immigration from non-EU countries is closely linked to labour demand rather than being treated as a free process. Each year, the government establishes admission quotas for foreign workers through the *decreto flussi* system, with the objective of addressing labour shortages while maintaining state control over migration flows. This approach reflects a view of migration as both an economic resource and a phenomenon requiring strict regulation.

The prevention of irregular migration constitutes a second key pillar of Italian immigration policy. The legal framework includes measures aimed at preventing unauthorised entry and residence, with irregular migration subject to administrative sanctions and, in some circumstances, criminal penalties. Enforcement mechanisms include sanctions against those facilitating irregular migration, border rejection procedures, administrative expulsions, and removals on public security grounds.

Administrative expulsion remains the principal enforcement instrument. Foreign nationals may be expelled when there is a risk of absconding or where applications for residence permits are found to be fraudulent. When immediate removal is not feasible, migrants may be allowed voluntary departure and access to assisted return programmes. Over time, the legal framework has also incorporated provisions addressing human trafficking, labour exploitation, and modern slavery, introducing protection measures for victims who cooperate with the authorities.

The third component of the system concerns migrant integration. Italian legislation guarantees a broad range of rights to legally resident foreign nationals and promotes their participation in social life. Fundamental constitutional rights, including personal liberty, equality before the law, and access to justice, apply equally to citizens and migrants. The Constitution also recognises the right to asylum, reinforcing Italy’s obligations under international and European human rights frameworks.

Citizenship represents one of the principal instruments of integration. It may be acquired through birth, marriage, or naturalisation. Naturalisation generally requires 10 years of continuous legal residence and remains subject to administrative discretion. Italian citizenship is regarded as the highest level of integration because it grants full participation in the political and legal community, including voting rights and access to public office.

The integration framework also includes anti-discrimination measures designed to protect migrants and promote equal opportunities. Italian legislation prohibits discrimination based on race and ethnic origin, while additional provisions derived from European Union law ensure equal treatment in employment and other social domains. The framework further guarantees access to essential social rights. Family reunification policies support family unity, while special protections are provided for minors. Healthcare and education are generally accessible regardless of nationality, and healthcare services are often available even to undocumented migrants.

Additional support is provided through housing and social assistance programmes. Local authorities, regional governments, and civil society organisations offer reception services, temporary accommodation, and support for migrants facing economic hardship. Long-term residents and many regular migrants may also access welfare programmes, reflecting an institutional commitment to social inclusion.

Despite these protections, the evolution of Italian migration policy has been marked by a persistent emphasis on migration control. Sciortino and Vittoria (2023) identify two main phases in the development of Italian migration policy between 1986 and 2019. The first phase (1986–2008) was characterised by a tension between labour market demand and restrictive admission policies. Legal entry channels proved insufficient to meet employers’ needs, leading governments to implement repeated large-scale regularisations (*sanatorie*) for migrant workers already employed in the informal economy.

The second phase (2008–2019) was characterised by a growing emphasis on border control and the securitisation of migration. During this period, migration was increasingly framed as a security issue, particularly within populist political discourse. Consequently, public policy focused more heavily on controlling mobility and combating irregular migration than on promoting long-term integration.

As a result, although Italy hosted almost five million foreign residents by 2019, integration policies remained relatively underdeveloped. While the legal framework formally guarantees extensive civil and social rights, migration policy continues to focus primarily on regulating entry and addressing irregular migration. The Italian immigration model can therefore be described as stable but restrictive, combining significant integration rights with strong control mechanisms and a limited long-term strategy for the social and political inclusion of migrants and their descendants.

Ambrosini and Campomori (2024) provide an insightful analysis of migration policies in the Italian case. Based on the distinction between immigration policies and policies for immigrants, the text points out the paradoxes characterising this field (the critical distance between expectations and outcomes; the tension between integration and exclusion). The authors recognize the nexus between migration flows and irregular economic activities, while pointing out the limits of *ex-post* interventions. A key point of the analysis is represented by the possibilities of integrating multilevel governance modalities, giving room to local actors to cope with the multiple issues at stake. Guidi and Berti (2023) documented phenomena of labour exploitation in Tuscan agriculture despite the formal legality of recruitment contracts. Geddes and Pettrachini (2020) showed that the persistent demand for labour in many sectors of the Italian economy tends to attract undocumented migrants in a context of increasing political pressure to control immigration. In summary, the principal source of migrant vulnerability within the Italian system is not simply the absence of legal rights. Rather, it lies in the tension between a rights-based integration framework and a migration regime primarily oriented towards control. Therefore, the following proposition is advanced:

Proposition 1: *Restrictive entry channels, combined with labour market dependency and insufficient integration policies, create conditions under which migrants may become economically, socially, and legally vulnerable*.

The planning of migrant admissions, the emphasis on controlling irregular immigration, and the relatively limited level of integration support constitute systemic conditions that may contribute to migrant vulnerability, despite the inclusionary aspirations embedded in anti-discrimination measures. Together, these factors represent the first building block of the theoretical interpretation proposed in this study.

This does not mean that the vulnerability of foreign workers is a direct consequence of these factors alone. Rather, the mechanism through which they generate vulnerability lies in their interaction with the large number of migrants seeking employment. An abundant labour supply enables farms and agricultural firms to benefit from competition among workers. At the same time, restrictions on entry and limited integration opportunities reduce migrants’ capacity to participate effectively in labour market processes and to exercise bargaining power. As a result, their vulnerability within the employment relationship is likely to increase^3^.

#### 3.1.2 The impact of legislation on vulnerability

Vulnerability is increasingly understood as a multidimensional condition resulting from both exposure to risks and a limited capacity to cope with their consequences. In particular, the factors identified in Proposition 1 may trigger institutional practices that have significant effects on vulnerability. Exposure to risk is influenced by the absence or inadequacy of rules designed to protect migrants or prevent the emergence of various forms of risk. Similarly, the capacity to cope with the consequences of risk is shaped by the effectiveness of institutions that support individuals in managing uncertainty and adverse conditions (North, 1990; Acemoglu and Robinson, 2012). Accordingly, Busetta et al. (2021) define vulnerability as a condition of heightened exposure to risk combined with a reduced ability to protect oneself and respond effectively. Their framework identifies several interconnected dimensions of vulnerability, including poor physical and subjective health, inadequate housing conditions, irregular legal status, low educational attainment, and exclusion from employment. Building on this perspective, Mendola et al. (2026) argue that vulnerability is not merely an individual characteristic but is produced through the interaction between migrants and the institutional environment of the host country.

In the Italian context, legal precarity and the resulting legal uncertainty emerge as major sources of vulnerability, restricting access to rights and social protection. At the same time, insufficient integration may generate economic hardship, particularly food insecurity, thereby deepening exclusion and reducing resilience. While many migrants report relatively good physical health, mental health outcomes are often significantly worse. Moreover, discrimination, racism, and broader processes of social exclusion may reinforce migrants’ vulnerability despite their efforts to integrate into society (Mendola et al., 2026).

Jacobsen (2024) further argues that states themselves may contribute to the production of vulnerability through forms of institutional abandonment and destitution, which can in turn generate insecurity and exclusion. Likewise, insufficient integration and legal precarity may weaken the effective enforcement of labour rights, increase dependence on restrictive employment arrangements, and limit access to pathways towards permanent residence, ultimately contributing to social exclusion (Lenard and Straehle, 2010). In this context, inadequate enforcement of labour rights plays a particularly significant role. Similarly, Marsden (2012) highlights how a “precarious migration status” generates differentiated forms of vulnerability through legally constructed hierarchies among migrant workers.

Li and Whitworth (2016) show that institutional arrangements in Australia can facilitate the exploitation of temporary migrants and advocate stronger labour inspections together with a clearer separation between employment rights and immigration status. Silverman and Hari (2016), examining Canada’s Seasonal Agricultural Worker Program, argue that workers’ choices are constrained by structural conditions that severely limit their alternatives. Taken together, these studies suggest that restrictive migration regimes and precarious legal statuses are key institutional factors shaping migrant vulnerability and labour exploitation (Lenard and Straehle, 2010; Marsden, 2012; Li and Whitworth, 2016; Silverman and Hari, 2016; Jafari et al., 2024).

The literature therefore suggests that the institutional context plays a crucial role in transforming the conditions identified in Proposition 1 into actual forms of vulnerability. In other words, the conditions created by the legal and institutional framework may significantly influence the degree of vulnerability experienced by migrant workers. Accordingly, the following proposition is advanced:

Proposition 2: *Institutional practices that shape access to rights, protection, and social inclusion contribute to determining the degree of vulnerability experienced by individuals employed in the agricultural sector*.

The focus on institutional practices highlights a key aspect of the analysis proposed here. Beyond the legal framework itself, institutional practices shape the opportunities available to migrants and influence the process through which they become foreign workers. For example, expanding the range of recruitment opportunities or ensuring recruitment under stronger labour-rights protections (Carchedi, 2020) reduces the risk of vulnerability. Similarly, the design and effective implementation of labour contracts can contribute to reducing migrants’ exposure to exploitative conditions.

### 3.2 Illegal labour mediation

The second building block of this study concerns the role of illegal institutions in mobilising labour resources within the agricultural sector. Illegal labour mediation represents the principal informal and illegal institution characterising the recruitment process. The following section first examines its main features and then discusses the limitations of Italian law enforcement mechanisms.

Recent research increasingly interprets migrant labour exploitation in agriculture as the outcome of interconnected migration, labour market, and agrifood systems rather than as a series of isolated abuses.

Perrotta and Raeymaekers (2023) argue that caporalato in Southern Italy should be understood as a form of “caporalato capitalism.” Illegal labour intermediaries do not merely recruit workers; they also provide transportation, housing, food, and labour organization, thereby externalising the costs of social reproduction from firms and public institutions onto migrant communities. This process generates the “adverse incorporation” of migrants into global supply chains, whereby workers are economically indispensable yet socially marginalised. The growing “refugeeisation” of agricultural labour has further intensified this dynamic, as asylum seekers and refugees constitute a highly flexible workforce. The authors also criticise Law No. 199/2016 for focusing on criminal brokers while neglecting the structural foundations of exploitation.

A broader perspective is offered by Tagliacozzo et al. (2024), who propose a systems-thinking approach to migration. They argue that migration interacts with multiple societal systems, including labour markets, agrifood production, healthcare, and disaster management. Global shocks such as COVID-19, Brexit, and the war in Ukraine reveal complex interdependencies characterised by multiple mechanisms are involved. For the purposes of this study, particular attention is devoted to *spillover and cascading effects*, as these may generate unintended consequences that affect systems or actors not originally targeted by a policy. While spillovers refer to broader indirect effects, cascading effects trigger a sequence of events that progressively intensify as they spread across different systems. Particular attention is also paid to *system intersectionality*, which refers to the way in which interconnected social identities, such as race, gender, and legal status, interact to shape an individual’s specific experience of vulnerability at the intersection of multiple systems.

Similarly, Melossi (2021) analyses the exploitation of Sub-Saharan tomato pickers in Puglia through the concept of differential inclusion. Migrants are subjected to processes of racialisation, criminalisation, refugeeisation, and deportability, while being segregated in informal settlements or “ghettos.” Exploitation is linked to the “retail revolution,” whereby supermarket purchasing strategies exert downward pressure on prices, which is then transmitted through the supply chain to workers via the “caporalato” system. Melossi argues that legal repression alone cannot address the structural causes of exploitation.

Lewis et al. (2015) develop the concept of *hyper-precarity* to explain how restrictive immigration regimes and flexible labour markets interact to create severe forms of exploitation. Rejecting a strict distinction between free and forced labour, they propose a “continuum of unfreedom” in which migrants, particularly asylum seekers and refugees, experience heightened vulnerability owing to their exclusion from employment and welfare systems. Legal insecurity and deportability thereby become mechanisms that sustain labour exploitation.

Omizzolo (2020) analyzes Italian labour exploitation as a structural, rather than exceptional, component of global capitalism. He introduces the concept of “refugeeization” (“profughizzazione”), where the state—specifically through the Security Decree (Law 132/2018)—intentionally traps refugees in legal precariousness to ensure a supply of “blackmailable” labour. Empirical evidence gathered in the Indian community in Agro Pontino reveals a “juridification” of exploitation: formal contracts and payslips are used to mask “grey work” and systematic rights violations. This system is maintained by a criminal network involving both “caporali” and “white-collar” professionals like accountants and public officials. Workers agency is emphasised, because despite extreme vulnerability, the communities investigated have organize collective resistance.

Comparative studies demonstrate that dependence on migrant labour exists across different institutional contexts. Corrado et al. (2024) compare Italy and Sweden and find that both countries rely heavily on migrant workers despite their contrasting migration regimes. Labour shortages are often politically constructed, reflecting employers’ preferences for a flexible and low-cost workforce. The authors argue that labour shortages are “politically constructed” rather than inevitable, often reflecting employers’ preference for a readily available and exploitable labour force capable of maintaining market competitiveness. Although the two countries adopt markedly different regulatory approaches, with Italy characterised by formal restrictions and the “refugeeisation” of agricultural labour, and Sweden by a liberalised, employer-led system, both demonstrate a structural dependence on migrant workers. The study highlights an ongoing shift towards a “corporate-environmental food regime,” in which discourses of sustainability and food quality increasingly influence migration policymaking. In addition to political constructions, refugeeisation, and legal system effects, spillover effects linking migration and labour policies also emerge as important mechanisms.

In the Italian context, Ambrosini (2024) employs the concept of *subordinate integration* to describe how migrants are economically incorporated into low-status occupations while remaining socially marginalised. He identifies four patterns: severe exploitation based on informal employment and *caporalato*; regulated seasonal employment; stable but socially devalued occupations; and a smaller segment of skilled employment. These labour arrangements are closely associated with different forms of housing insecurity and social exclusion.

Research conducted in other countries reveals similar dynamics. Fauser and Linsel (2026) show how German media narratives legitimise precarious seasonal agricultural work through the construction of the “good migrant worker,” while obscuring the transfer of social reproduction costs to workers’ countries of origin. Keegan (2024) argues that the United States H-2A program creates a condition of “liminal legality”, granting employers substantial control over migrant workers while denying them full citizenship rights. Such arrangements facilitate wage theft, unsafe working conditions, and the reproduction of racialised labour hierarchies.

Other studies broaden the analysis beyond international migration. Deshingkar et al. (2012) find that internal migration remains largely neglected in labour and development policies despite its importance for poverty reduction. Internal migrants are often concentrated in informal sectors, including agriculture, where labour rights are weakly enforced. Millán-Quijano and Pulgarín (2023) demonstrate that the expansion of Colombia’s palm oil sector contributed to forced internal displacement through alliances between agribusiness interests and paramilitary groups in a context of weak property-right institutions. Similarly, Smith (2015), examining Canada’s Seasonal Agricultural Worker Program, shows how local opposition to migrant housing reproduces racialised forms of labour control and reinforces workers’ social exclusion.

Overall, these studies converge on the idea that migrant labour exploitation is structurally produced through the interaction of migration policies, labour market institutions, agrifood supply chains, and broader social relations (Lewis et al., 2015; Melossi, 2021; Perrotta and Raeymaekers, 2023; Corrado et al., 2024; Ambrosini, 2024; Tagliacozzo et al., 2024; Keegan, 2024; Fauser and Linsel, 2026).

Based on the previous analysis, the following proposition can therefore be advanced:

Proposition 3: *The consolidation and evolution of illegal recruitment systems have, on the one hand, expanded their recruitment capacity through the involvement of intermediaries drawn from the same ethnic communities as the workers and, on the other hand, appear capable of linking workers’ legal vulnerability to their wage levels*.

The Table 1 shows that all the mechanisms identified in the literature are implemented through a combination of different types of institutions. Of course, all mechanisms entail the involvement of farmers, employers and workers. However, some mechanisms operate very general system level, whereas others primarily affect the rights and working conditions of individual migrants.

**Table 1:** Mechanisms of migrant labour exploitation in agriculture.

Mechanism	Description	Main Effect	Main Authors
a) Benefits for farms and food companies			
Externalization of social reproduction costs	Gangmasters provide housing, transport, food and labour organization, shifting reproduction costs from firms and public institutions to migrants.	Lower labour costs and greater worker vulnerability.	Perrotta and Raeymaekers (2023)
Agrifood supply-chain pressures	Retailers impose downward price pressures along supply chains.	Cost reduction through labour exploitation.	Melossi (2021)
Transnational labour arbitrage (between Germany and other countries)	Narrative illustration of differences between country of origin and country of working	Conceals externalization of social reproduction costs.	Fauser and Linsel (2026)
b) Weakening labour rights			
System interdependencies	Migration interacts with labour markets, agrifood systems, welfare and governance through feedback loops.	Spillover and Cascading Effects Intersectionality	Tagliacozzo et al. (2024)
Adverse incorporation	Migrants are economically indispensable but socially marginalised.	Economic integration without social inclusion.	Perrotta and Raeymaekers (2023)
Differential inclusion	Migrants are incorporated through racialization, criminalization, “refugeization” and deportability.	Employment combined with exclusion.	Melossi (2021)
Synergies between labour market flexibility and restrictive migration regulation	Restrictive migration regimes interact with flexible labour markets.	Hyper-precarity with consequential extreme vulnerability and exploitation Compromised citizenship rights	Lewis et al. (2015)
Political constructions, “refugeeization” system effects due to the laws, spillover effects connecting migration and labour policies.	Institutions restrict labour supply while employers seek cheap labour.	Structural dependence on migrant workers	Corrado et al. (2024)
Subordinate integration	Migrants concentrate in low-status jobs.	Occupational segmentation and marginalization.	Ambrosini (2024)
Housing insecurity	Labour exploitation is linked to precarious housing conditions.	Reinforcement of social exclusion	
“Refugeeization” of agricultural labour	Refugees and asylum seekers become a highly flexible workforce.	Expansion of vulnerable labour supply	Perrotta and Raeymaekers (2023); Lewis et al. (2015); Dines (2023); Corrado et al. (2024)
Selective inclusion	Temporary worker programs grant work access without full rights.	Legal status, but limited citizenship rights	Keegan (2024)
Weak labour-rights protection	Migrant workers often operate in poorly regulated contexts.	Higher exposure to exploitation	Deshingkar et al. (2012)

Source: the author.

At the system level, *Benefits for farms and companies* are high-powered incentives generated by the system of illegal practices. The externalisation of social reproduction costs requires not only illegal institutions, which can nevertheless operate effectively when there is a demand for labour, but also formal organisations that channel such demand, often obscuring actual labour relations through formally lawful contractual arrangements. This process results in the internalisation of benefits by farms and food-processing companies.

Similarly, the pressure exerted by large-scale retail distribution operates within the framework of formally regulated (European) markets. However, it interacts with mechanisms of labour exploitation that may also emerge in legally regulated contexts (Guidi and Berti, 2023; Carchedi, 2020; Mirabella et al., 2026), rather than with mechanisms aimed at generating additional value (Marchini et al., 2016; Masi et al., 2025).

This line of argument, however, tends to understate the fact that the organisation and allocation of labour within firms are also influenced by firms’ own levels of efficiency. These outcomes are not solely determined by bargaining power relations along the supply chain but are also the result of managerial decisions that, at least in principle, may be taken independently of such relations (Marchini et al., 2024).

In Table 1, the label *Weakening Labour Rights* refers to mechanisms that are likely to directly affect migrants’ rights. These mechanisms operate by limiting inclusion, increasing labour market flexibility, heightening vulnerability, and weakening the legal protection of workers’ rights. These involve formal institutions which can trigger the transformation of the weakness conditions into economic value only through the operation of informal institutions that facilitate workers’ mobility across sectors or segments of the labour market; sustain social and discursive mechanisms that discourage resistance to labour intermediaries (*caporali*) (Poppi, 2025); foster forms of social interaction rooted in ethnic affiliation (Carchedi, 2020, 2024); and reinforce workers’ isolation from local communities. The interdependence between labour markets, recruitment processes and governance (Tagliacozzo et al., 2024) arrangements is associated with increasing marginalisation and labour flexibilisation. At the same time, a significant set of individual rights is progressively eroded (see among others Perrotta and Raeymaekers (2023); Lewis et al. (2015); Dines (2023); Corrado et al. (2024), leading to the emergence of a workforce willing, or compelled, to accept any form of recruitment and employment arrangement.

### 3.3 Law and enforcement

Regulations concerning illegal labour are stringent in many countries. However, the literature shows that, in practice, enforcement is often more important than the law itself. Pignatti (2026) finds that increasing the number of labour inspectors in Colombia significantly improves compliance among formal firms, resulting in more written contracts, greater pension coverage, and a reduction in excessive working hours. However, informal firms remain largely unaffected because they are difficult to identify and monitor. The study suggests that stronger enforcement does not necessarily reduce employment, particularly where formal employers possess monopsony power.

Menashe (2026) argues that the International Labour Organization (ILO) exerts influence not through direct sanctions but through its role as a global normative authority. ILO standards increasingly shape trade agreements, corporate responsibility initiatives, and supply-chain governance, thereby creating incentives for compliance and promoting the harmonisation of labour rights at the global level. Brotherhood et al. (2024), examining the Brazilian case, show that labour inspections can effectively reduce informality when firms face penalties and are required to formalise workers. The authors emphasise that enforcement outcomes depend on inspection design, targeting strategies, and the use of firms’ compliance records. Alfano et al. (2026) demonstrate that Italy’s *White List* certification scheme, which signals freedom from mafia influence, improves firms’ productivity and profitability, particularly among small enterprises operating in high-risk sectors. The certification functions as a reputational mechanism that strengthens trust and reduces information asymmetries. Although this type of certification is not currently available in the agricultural sector, the underlying institutional mechanism highlights the complementary role of certification schemes in fostering a reputation for legality. This, in turn, may generate significant incentives supported by consumer preferences and market demand. Finally, Koukiadaki (2024) highlights the fragmented nature of labour-law enforcement within the European Union. While the Court of Justice of the European Union has strengthened workers’ access to remedies, significant enforcement gaps remain. The study therefore calls for a dedicated EU directive establishing common enforcement standards and more effective mechanisms for worker protection.

Overall, the literature suggests that effective enforcement requires a combination of inspections, reputational incentives, legal remedies, and institutional coordination, while remaining constrained by the persistence of informality and broader governance weaknesses.

Italian legislation against *caporalato* (illegal labour intermediation and labour exploitation) is primarily based on Article 603-bis of the Criminal Code, substantially strengthened by Law No. 199/2016. The reform expanded criminal liability by targeting not only illegal intermediaries (*caporali*) who recruit workers under exploitative conditions but also employers who knowingly benefit from such practices. The law provides severe sanctions, including imprisonment, fines, confiscation of assets, preventive seizure measures, and judicial supervision of firms. It also combines repression with worker protection through assistance programmes, support for vulnerable workers, and stronger coordination among labour inspectors, law-enforcement agencies, and social services. Preventive initiatives, such as the *Rete del Lavoro Agricolo di Qualità*, were introduced to encourage lawful recruitment practices and increase transparency within agricultural labour markets.

Despite being one of the most comprehensive frameworks against labour exploitation in Europe, enforcement remains challenging. Labour exploitation often occurs in hidden and informal contexts, including farms, warehouses, and subcontracting networks, making detection difficult. Many victims are migrants, seasonal workers, or undocumented individuals who fear dismissal, retaliation, or immigration-related consequences and are therefore reluctant to cooperate with the authorities. Prosecutors must demonstrate not only the existence of irregular employment practices but also exploitation, workers’ vulnerability, and a causal relationship between recruitment and abuse, creating substantial evidentiary challenges.

Additional obstacles arise from complex supply chains that obscure responsibility, limited inspection resources, and strong economic incentives encouraging employers to reduce labour costs through illegal practices. Regional and sectoral differences further complicate enforcement and require targeted interventions. Moreover, lengthy criminal proceedings may weaken the deterrent effect of sanctions, while preventive mechanisms remain underutilised. Consequently, the effectiveness of the Italian framework depends not only on criminal penalties but also on labour inspections, victim protection, lawful recruitment channels, and coordinated action among labour, judicial, police, and social-welfare authorities (see Table 2).

**Table 2:** Mechanisms of labour enforcement and compliance.

Mechanism	How it works	Expected effect	Key references
Labour inspections	Monitoring firms and detecting violations through workplace inspections.	Greater compliance, formalisation of employment, improved working conditions.	Pignatti (2026); Brotherhood et al. (2024)
Sanctions and criminal liability	Fines, imprisonment, confiscation of assets, and judicial supervision.	Increased deterrence and reduced incentives for illegal labour practices.	Law No. 199/2016; Art. 603-bis
Targeted enforcement	Risk-based inspections and compliance records.	More effective detection and enforcement outcomes.	Brotherhood et al. (2024)
Normative pressure	International labour standards influence behaviour without direct sanctions.	Voluntary compliance and harmonisation of labour rights.	Menashe (2026)
Reputational incentives	Certification schemes signal legal compliance.	Higher trust, productivity, and lawful behaviour.	Alfano et al. (2026)
Legal remedies for workers	Access to courts and rights-claiming mechanisms.	Stronger worker protection and accountability.	Koukiadaki (2024)
Worker protection programmes	Support services for vulnerable workers.	Greater reporting of abuses and victim recovery.	Law No. 199/2016
Lawful recruitment channels	Transparent recruitment and intermediation systems.	Reduced dependence on caporali.	Rete del Lavoro Agricolo di Qualità
Institutional coordination	Cooperation among inspectors, police, courts, and social services.	Improved detection and support.	Koukiadaki (2024); Law No. 199/2016
Structural constraints	Informality, migrant vulnerability, fragmented supply chains.	Reduced enforcement effectiveness.	Pignatti (2026); Koukiadaki (2024); Menashe (2026)

Source: the author.

It can therefore be argued that:

Proposition 4: *The effectiveness of legislation against illegal labour intermediation depends on the institutional capacity to implement and enforce legal provisions through inspections, coordination, victim protection, and supply-chain accountability.*

## 4 An institutional perspective

### 4.1 Interaction between formal and illegal institutions

As mentioned above, this study understood agricultural labour exploitation as a set of practices through which employers or labour intermediaries derive economic benefits from violations of labour regulations and workers’ rights (Carchedi, 2020, 2024; Guidi and Berti, 2023; Torre, 2018). Exploitation is then held as a multidimensional process involving employers, intermediaries, institutions, and market forces. The restructuring of agrifood value chains and the market power of large retailers push producers to rely on highly flexible and underpaid labour managed through illegal intermediaries. She also highlights the state’s role in creating migrant vulnerability through legal and administrative practices that foster precariousness. Effective solutions therefore require addressing the economic and institutional roots of exploitation rather than focusing solely on individual criminal responsibility. Although this interpretation provides a valuable understanding of the exploitation process, it does not fully explain the institutional mechanisms that generate and sustain it.

Illegal institutions such as *caporalato* constitute a specific subcategory of informal institutions, consisting of rules and organisations that actively violate the law. The key issue, therefore, concerns the relationship between the illegal system of rules and practices (Perrotta and Raeymaekers, 2023; Corrado et al., 2024) and the formal institutions that regulate the labour market, migration flows, and, to some extent, the agricultural sector itself. This study argues that these relationships can be analysed through the distinction between formal and informal institutions (North, 1990) and through the transformative effects that their interaction may exert on economic systems (North, 1990; Acemoglu and Robinson, 2012; Shirley and Xu, 2025; Shirley, 2026).

Hodgson (2025) argues that the most useful distinction between formal and informal institutions concerns the *means of enforcement* rather than whether rules are simply “written down.” He defines formal institutions as those constituted or maintained through legally prescribed procedures and potentially enforced by the state. Informal institutions, by contrast, are systems of socially embedded rules that operate outside the legal system. They are typically enforced by peers or social groups that impose social costs on individuals who fail to comply with them. Examples include customs, traditions, language, etiquette, and social conventions. Hodgson (2025) notes that while informal institutions are non-legal, they are not necessarily illegal, although illegal organisations such as mafias also fall within this category. He further explores the interaction and evolution of these institutional systems, arguing that legislation may sometimes act as a “magnet,” rapidly drawing informal norms towards new legal standards.

Based on Helmke and Levitsky's (2004) taxonomy, the illegal institutions discussed here should be considered competing institutions because:

- They are not complementary, since the outcomes generated by illegal practices diverge from those intended by formal institutions;
- The effectiveness of formal institutions is limited, making it unlikely that illegal practices can be classified as accommodating institutions.

More precisely, informal institutions structure behaviour in ways that are incompatible with formal rules. As a result, compliance with one set of rules tends to entail the violation of another (Helmke and Levitsky, 2004, p. 729). Table 3 summarises the proposed classification.

**Table 3:** Classifying the illegal institution aimed at labour exploitation.

Outcomes	Effective formal institutions	Ineffective formal institutions
Convergent	Complementary	Substitutive
Divergent	Accommodating	Competing (Caporalato and other illegal system of practices)

Source: elaboration of the author based on Helmke and Levitsky (2004).

However, this conclusion requires further qualification. The interaction among institutions of different types can itself be characterised as *competing* in nature. This circumstance has two important implications. First, even when interaction is not explicitly conceived in evolutionary terms (Hodgson, 2025), it inherently implies that behaviour is shaped by both sets of institutions involved. Consequently, competition between alternative systems of rules becomes a defining feature of the socio-economic system in which they operate. As a result, the orientation and objectives of one system of rules, even when it does not prevail over the other, nevertheless contribute to shaping the overall characteristics of that system.

Second, this competing interaction may have significant effects on entrepreneurial behaviour. A plausible outcome is that entrepreneurs gradually adjust their actions to the incentives created by illegal institutions, thereby aligning their behaviour with the opportunities and constraints generated by those institutions (Martino, 2018).

### 4.2 An integrative framework

The analysis undertaken so far highlights the distinctive nature of institutional interaction in the case under investigation. A useful way to substantiate the proposed theoretical framework is to clarify the relationships among the key dimensions of migrant labour exploitation in Italian agriculture, namely the regulatory framework, vulnerability, and illegal labour intermediation, and the theoretical propositions derived from the literature. Figure 1 provides a diagrammatic representation of these relationships.

**Figure 1:**
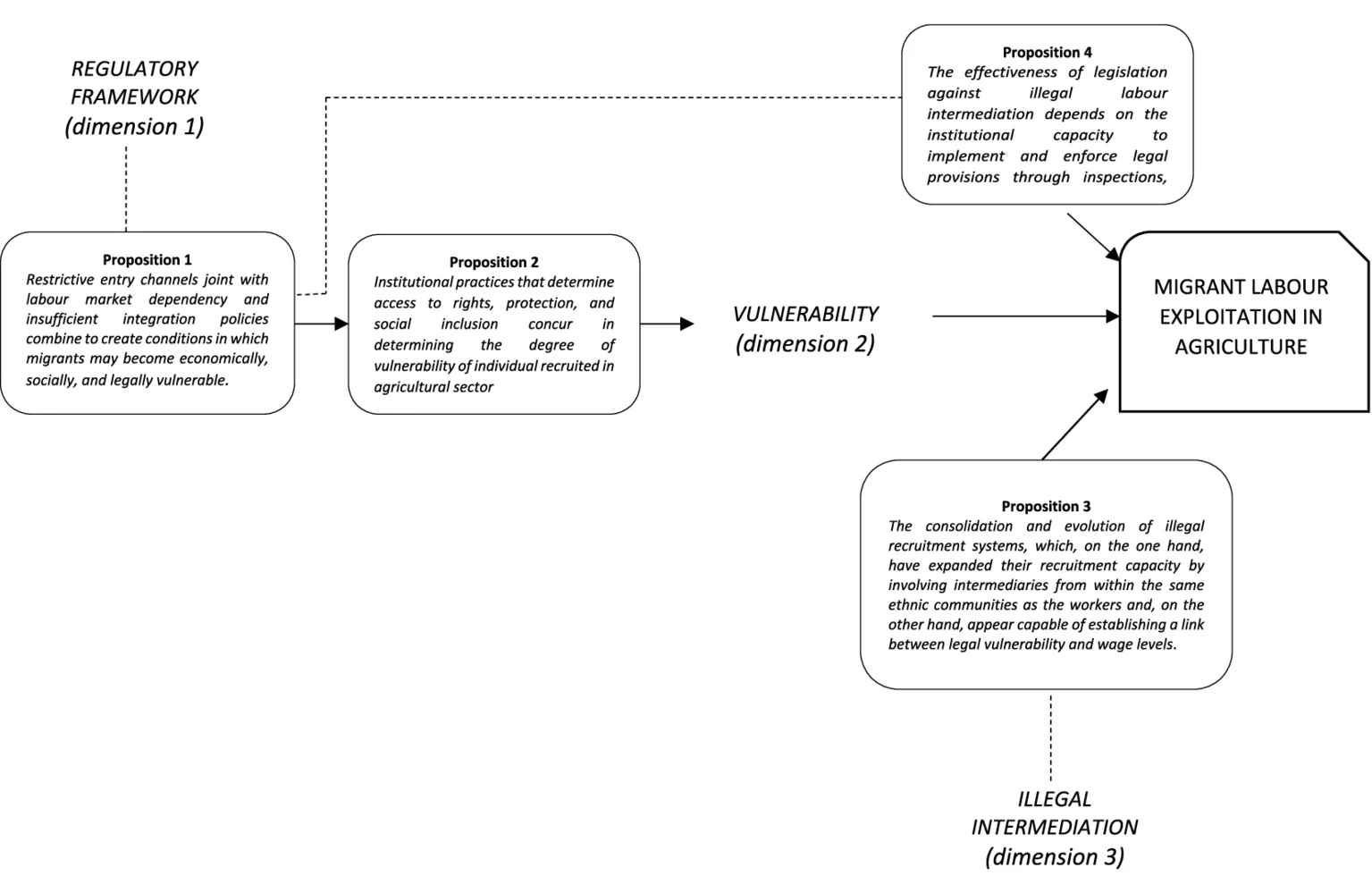
An integrative theoretical framework. Source: the author.

The dashed lines connecting the regulatory framework to Proposition 1 and illegal labour intermediation to Proposition 3 indicate that these dimensions constitute features of the socio-economic system and can therefore be conceptualised and delineated through institutional theory. In contrast, the relationship between Proposition 1 and Proposition 2 highlights how formal rules and their degree of enforcement are mediated by a set of institutional practices which, while legally legitimate, reflect the incomplete protection of rights and the weak social inclusion of migrant workers. These practices generate conditions of vulnerability which function as a transmission mechanism between institutional arrangements and labour-market outcomes.

The literature reviewed supports the view that these institutional practices underlie migrants’ limited access to inclusion processes, services, civic participation, and long-term integration. The theoretical framework proposed here therefore incorporates migrants’ conditions of vulnerability and exclusion into the process of institutional interaction, and rejects the notion that these conditions are the result of isolated or incidental circumstances.

Labour exploitation emerges from this condition of vulnerability through the interaction between illegal recruitment practices and the ineffective enforcement of existing regulations. Vulnerability therefore plays a pivotal mediating role, linking institutional configurations to exploitative outcomes and shaping the incentives and constraints faced by economic actors.

Several findings from the previous analysis support this interpretation:

- Vulnerability originates from the internal tensions and contradictions embedded within the regulatory framework (Propositions 1 and 2);
- Illegal recruitment systems have evolved and consolidated over time, expanding their recruitment capacity through the involvement of intermediaries drawn from the same ethnic communities as the workers and establishing a link between legal vulnerability and wage levels (Proposition 3);
- Formal institutions remain incomplete, as evidenced by the shortage of resources available to the authorities responsible for inspections and monitoring activities required to enforce Law No. 199/2016 effectively (Proposition 4).

The development of this competing interaction between formal and illegal institutions therefore provides a plausible explanation for the research question addressed in this study. According to the theoretical perspective proposed here, varying degrees of labour exploitation may emerge as a consequence of institutional interactions. Even where workers enjoy formal legal protection, the potential for exploitation may persist because the availability of vulnerable workers tends to weaken bargaining power within the labour market as a whole.

This does not imply, of course, that all farmers exploit the workers they employ. However, as illegal institutions are able to trigger exploitative behaviours, the theory suggests that exploitative outcomes are expected to occur wherever competitive interactions between formal and illegal institutions arise. The prevalence of exploitation may depend on the intensity of these interactions; however, some degree of exploitation is nevertheless expected to occur. Consequently, the theory could be tested and potentially falsified by identifying cases in which competitive interactions between formal and illegal institutions are present but exploitative outcomes do not occur.

### 4.3 Discussion

At least two lines of research on the exploitation of agricultural and migrant labour can be identified In the literature. These lines differ, among other respects, in their epistemological orientation.

The first line of research, exemplified by Corrado and Caruso (2022), Abbatecola et al. (2022), and Palumbo et al. (2022), is characterised by the formulation of general assumptions that recognise the existence, within European and other economic systems, of a specific labour regime and a particular capitalist configuration of those systems, sometimes understood more specifically as neoliberal capitalism. These studies presuppose labour exploitation as an inherent characteristic of the economic system: the facts they analyse are essentially compared against a theory of capitalism. From an epistemological perspective, therefore, such research appears to be primarily aimed at assessing empirical evidence in relation to an existing theoretical framework (Kuhn, 1970, pp. 25 ff.).

A second body of research, represented, for example, by Guidi and Berti (2023), Salvia (2024), and Perrotta and Raeymaekers, 2023, does not rely on such strong assumptions. Instead, it develops the analysis of the phenomenon by illuminating its various dimensions, thereby contributing to a progressively more comprehensive understanding of it and fully recognising the novelty that these phenomena display from economic, social, and historical perspectives. The present study seeks to position itself within this second research tradition. From an epistemological standpoint, this approach aims to elaborate the theory of economic institutions (Kuhn, 1970, pp. 25 ff.) and to advance theoretical developments whose validity remains open to assessment and whose potential usefulness, from a pragmatic perspective, may contribute to public decision-making processes.

The analysis undertaken has made it possible to highlight the logical and empirical relationships between the modes and levels of effectiveness of regulatory intervention and the illegal practices involved in the recruitment of foreign workers. Carchedi (2020, 2024), Omizzolo (2020), and Corrado et al. (2024) have extensively documented the impact of these relationships on the diffusion of labour exploitation in agriculture. The explanatory factors involved have been widely explored in the literature, although their interaction has not, until now, been systematically examined. The innovative contribution of the present study lies precisely in recognising the interaction between formal and informal institutions as a general and distinctive feature of the Italian case.

It should be emphasised that this study has focused on the interaction between formal and illegal institutions as a factor that may contribute to the diffusion of labour exploitation in Italian agriculture. The perspective adopted, therefore, does not address forms of labour exploitation that occur independently of illegal labour intermediation.

This is a theoretical contribution that is primarily grounded in the explicit introduction of the concept of institutions. Although the literature contains numerous studies highlighting the role of public policies in labour exploitation (e.g., Marsden, 2012), and the coexistence of private mechanisms (e.g., Corrado and Caruso, 2022) that produce comparable outcomes, what is lacking is an awareness that the set of institutions constitutes a fundamental cause of the observed phenomena.

This awareness can be achieved by redefining the theoretical relationships between the existing concepts and institutions (Figure 1), thus through a theoretical step made possible precisely by the concept of institution. The fact that institutions interact competitively reveals a structural feature of the economic system. Moreover, this step has practical implications that are easy to identify. For example, the competitive interaction identified here clearly indicates that weak law enforcement, on the one hand, is unequivocally a responsibility of the public sector and, on the other hand, represents a clear failure on the part of lawmakers to fulfil their role in countering illegal institutions.

The interaction between institutions implies their coexistence; however, such coexistence is not an inevitable outcome. For example, more extensive enforcement measures across the entire national territory, beyond extraordinary interventions (INPS, 2026), may reduce the capacity of illegal practices to compete with those promoted by legislation and may create the conditions necessary for legal norms to achieve their intended effects. Likewise, a policy approach placing greater emphasis on the protection of migrants’ rights and labour rights in agriculture would undoubtedly contribute to reducing the scope for competing interactions between formal and informal institutions.

Furthermore, several Italian regions have introduced legislation in this area with the specific aim of strengthening regulation and combating illegal practices. For instance, in 2006 the Apulia Region enacted Regional Law No. 28 of 26 October 2006 (Regione Puglia, 2006), aimed at tackling undeclared work and promoting labour legality. The law seeks to encourage the formalisation of informal employment through collaboration among public institutions, employers’ organisations, trade unions, and other social partners. It also promotes educational initiatives on legality and workers’ rights within secondary schools.

A central feature of the legislation is the link it establishes between access to public resources and compliance with labour regulations. Beneficiaries of regional subsidies, grants, public procurement contracts, and European, national, or regional funds are required to apply the relevant national and territorial collective labour agreements negotiated by the most representative employers’ and workers’ organisations. These obligations extend to contractors and subcontractors and remain in force throughout the period during which public support is received.

Similar strategic approaches, although implemented through slightly different institutional arrangements, have also been adopted by other Italian regions, including Tuscany, Emilia-Romagna, Calabria, Basilicata, Campania, and Sicily. More generally, an important role in this field may be played by the objective of social sustainability within the European Union’s Common Agricultural Policy, provided that this objective is maintained in future reforms (Canfora and Leccese, 2024).

The Quality Agricultural Labour Network (*Rete del Lavoro Agricolo di Qualità*) is an instrument established within the National Social Security Institute (INPS) with the aim of promoting legality in the agricultural sector and combating undeclared work, illegal labour intermediation, and labour exploitation. It was introduced by Decree-Law No. 91/2014 (converted into Law No. 116/2014) and was subsequently strengthened by Law No. 199/2016 on *caporalato*. Firms participating in the Quality Agricultural Labour Network are required to comply with labour, tax, and social security regulations, apply collective labour agreements, and maintain high standards of legality. In return, they benefit from enhanced reputation, greater market credibility, lower priority for labour inspections, and access to institutional networks and support initiatives.

Although there appears to be a market interest in products originating from firms belonging to the Network (Rossi et al., 2024), it is generally regarded as an important preventive tool, albeit one characterised by several limitations (Altamore et al., 2024). Among the most significant of these is the voluntary nature of firms’ participation. Membership requires entrepreneurs to assess whether the potential benefits associated with joining the Network outweigh the additional coordination and compliance costs that participation may entail.

Poppi (2025) showed how *caporali* develop justifications for their activities that may establish interpretative frameworks for foreign workers. This evidence is particularly relevant for understanding how illegal practices spread throughout agricultural supply chains. Beliefs form part of the cognitive rules that connect decisions to their outcomes (North, 2005; Greif and Mokyr, 2017). Consequently, efforts to influence belief formation are expected to shape incentives and guide actors in orienting their behaviour.

Conversely, at the level of culture as an informal institution (Hodgson, 2025), both the development of perspectives centred on solidarity and the diffusion of principles of legality may positively interfere with the mechanisms of competition between formal and informal institutions. Such cultural processes may therefore contribute to weakening the legitimacy of illegal recruitment practices and strengthening compliance with formal rules.

## 5 Conclusion

This study sought to identify the interaction between formal institutions and illegal (informal) institutions within labour recruitment processes in agriculture, with particular attention to migrant workers. Adopting a theoretical perspective, the analysis identified three key dimensions of this interaction: the incompleteness of the formal regulatory framework, individual vulnerability, and the persistence and evolution of illegal institutions. Although these factors have been discussed, at least partially, in the existing literature, their integration into a unified theoretical framework represents a novel contribution to knowledge in this field.

The perspective adopted focuses on specific institutional processes that may take different forms across contexts and may be revised in the light of new evidence capable of refining the proposed framework. Consequently, the study does not claim that its theoretical interpretation supports the hypothesis of a general transformation of agri-food supply systems or of the agricultural labour market. Nevertheless, the findings suggest that a more complex conceptualization of the agricultural labour market is necessary.

The analysis has also shed light on the multidimensional status of foreign workers subjected to exploitation. The literature clearly shows that this status must be understood in relation to legal precarity, discrimination, housing insecurity, exclusion from services, and subordinate forms of incorporation. This article has sought to connect these dimensions theoretically and to provide an integrated interpretative framework through which to understand them.

One of the main limitations of the study derives from the narrative approach adopted in the literature review. A systematic approach could better contextualise the insights drawn from the analysis. A further limitation derives from the case study itself and the challenges of generalisation. Interactions between formal and informal institutions can, of course, be found in different geographical contexts; however, the key point emphasised here is the illegal nature of the informal institutions under consideration.

A promising direction for future research is the development of empirical studies directly derived from the proposed theoretical framework. Such investigations could both test the validity of the interpretation advanced here and provide new insights into the complex realities under examination. Particular attention should be devoted to assessing the impact of the identified institutional interactions on firms and agri-food value chains. For instance, the widespread view that attributes a central role to large-scale retailers’ ability to depress agricultural commodity prices should be reconsidered in light of the apparently equally significant role played by the interaction between formal and illegal institutions.

## Data Availability

Publicly available datasets were analyzed in this study. This data can be found here: No data were used for this article. I only used scientific articles, all mentioned in the references list.

## Footnotes

1 *Institutional complementarity* is a broader concept. According to Aoki, institutional complementarity refers to a mutually reinforcing relationship among institutions, whereby the effectiveness of a particular rule or institutional arrangement depends on the presence of other compatible institutions. The concept highlights that institutional performance is shaped not by isolated institutions but by the coherence of broader institutional configurations. Institutional complementarity helps explain the diversity, stability, and path dependence of socio-economic systems, while also accounting for the difficulties and unintended consequences often associated with institutional reform (Landini and Pagano, 2026).

2 In this context, a rule is understood as an injunction or prescription stating that, under circumstances X, action Y should be performed (a positive rule) or action Z should not be performed (a negative rule). Rules may be constitutive (interpreting phenomenon X as Y) or regulative (governing actions and behaviour) (Hodgson, 2025, p. 14).

3 It should also be noted that a substantial body of literature highlights the difficulties experienced by agricultural enterprises in recruiting labour for production activities. The scope of this paper does not allow for a detailed examination of this argument. Nevertheless, it is important to recognise that official employment statistics cannot capture the contribution of labour recruited through illegal channels and, consequently, do not fully reflect the total labour supply, to use the terminology of economic analysis. See, for example, Win et al. (2025) and Hertz and Zahniser (2013).
